# Effects from supplementary feeding of bamboo powder in perinatal period on farrowing process, serum biochemical indexes, and fecal microbes of sows and offspring piglets

**DOI:** 10.3389/fmicb.2023.1139625

**Published:** 2023-04-27

**Authors:** Fawen Dai, Tao Lin, Xia Huang, Xiaolin Shi, Yaojun Yang, Xiang Nong, Jianjun Zuo, Hui Liu

**Affiliations:** ^1^College of Life Science, Leshan Normal University, Leshan, Sichuan, China; ^2^Key Laboratory of Bamboo Pest Control and Resource Development, Leshan, Sichuan, China; ^3^Guang’an Feed Industry Management Office, Guang’an, Sichuan, China; ^4^Beijing Vica Group Biotechnology Co., Ltd, Beijing, China; ^5^College of Animal Science, South China Agricultural University, Guangzhou, Guangdong, China

**Keywords:** bamboo powder, feed, sow, perinatal period, biochemical index, fecal microflora

## Abstract

**Introduction:**

This study was conducted to explore the effects of supplementary feeding of bamboo powder on the physical parameters of sows during the perinatal period of 7 days ± in parturition, including farrow duration, serum biochemical indexes, fecal physicochemical indexes, and microbial flora.

**Methods:**

Thirty pregnant sows were randomly divided into three groups: the control group was fed a basal diet, TRE1 group and TRE2 group were fed a basal diet supplemented with 30 g d^−1^ and 60 g d^−1^ bamboo powder, respectively. Multiple parameters of sows and offspring piglets were determined.

**Results:**

The contents of serum total cholesterol and triglyceride of sows in TRE2 group were significantly lower than those in the control group. The contents of serum malondialdehyde of sows in TRE2 and TRE1 groups were significantly lower than that in control group. The water content of sow feces in TRE2 group was significantly higher than that in control group, and the pH values of sows in TRE2 and TRE1 groups were significantly higher than that in control group. The richness index (Chao) of sow fecal bacterial community in TRE2 group was significantly lower than that of the control group, and the Ace and Sobs indexes tended to be lower than those of the control group. At the phylum level, the relative abundance of *Actinobacteriota* in the feces of sows in TRE2 group was significantly lower than that of the control group, while that of *Fusobacteriota* in the feces of suckling piglets in TRE2 group tended to be lower than that of the control group. At the genus level, among the Top10 dominant bacteria, the relative abundance of *Tissierella* in the feces of sows in TRE2 group was significantly lower than that of the control group while that of *Fusobacterium* in the feces of suckling piglets in TRE2 group tended to be lower than that of the control group. The relative abundance of *Clostridium_sensu_stricto_*1, *Terrisporobacter*, *Turicibacter*, and *Tissierella* in the feces of sows in TRE2 group was significantly lower than that of TRE1 group (*p* < 0.05), while *Lactobacillus* tended to be higher than that of TRE1 group (*p* < 0.10).

**Discussion:**

The results suggested that supplementary feeding 60 g d^−1^ bamboo powder could increase the water content in the feces of sows, reduce the oxidative damage, and tend to reduce the relative abundance of opportunistic pathogenic *Fusobacterium* for suckling piglets, while it reduced the fecal microbial diversity of sows.

## Introduction

Perinatal period of sows refers to the transition period from 7 to 10 days before delivery to 3–5 days after delivery. Although the perinatal period is short in the whole production cycle, it has an important impact on the production performance of sows. Many problems may occur in this period, such as constipation, prolonged farrowing duration of sows, and rising mortality rate of piglets (Theil et al., 2022). Constipation may cause discomfort to sows in the perinatal period, prolong farrowing duration, and increase intestinal absorption of endotoxin, leading to postpartum agalactia syndrome (Tabeling et al., 2003). Therefore, it may be helpful to solve the constipation of sows in the perinatal period by regulating fiber nutrition, thus improving production performance. For sows with postpartum agalactia syndrome, supplementary feeding dietary fiber in the perinatal period could significantly improve their feces score, postpartum agalactia syndrome score, body condition score, and farrowing performance (Papatsiros et al., 2021). In addition, some studies have found that increasing dietary fiber in the perinatal period of sows significantly reduced the number of stillbirths and total mortality of piglets, as well as the mortality rate and diarrhea rate caused by weak piglets (Feyera et al., 2017). Feeding high-fiber diet to sows during perinatal period could reduce *Clostridium perfringens* in feces, thus helping to improve the health of newborn piglets (Schwennen et al., 2022). All the above studies suggest that the fiber nutrition of perinatal sows may be significant to the health of both sows and their offspring piglets.

Fiber nutrition of sows plays an important role in regulating the intestinal microbial composition and the health status of the piglets. It was found that, elevating the dietary fiber level of sows with bran and soybean hulls as fiber sources significantly increased the microbial diversity of colonic chyme of piglets, and significantly improved the abundance of *Acidobacteria* and *Bacteroidetes* (He et al., 2020). Another study found that adding wheat bran and sugar beet pulp to the diet could significantly increase the abundance of *Lactobacillus* in the colon of piglets and the expression level of tight protein mRNA in the ileum (Shang et al., 2021b). One study compared with the effects of lignocellulose, modified cassava starch and konjac flour have significant effect on substrate fermentation. Konjac flour was a kind of fast fermentation fiber, which could produce propionic acid and butyric acid quickly. At 36 h of fermentation, the abundance of anaerobic *Anaerovibrio* and *Erysipelatoclostridium* was higher that of the konjac flour group, while at 72 h of fermentation, the abundance of *Fibrobacter* was higher in the lignocellulose group (Pi et al., 2021). *Fibrobacter* could degrade plant fibers with complex structure (Qi et al., 2005). The increased abundance of *Fibrobacter* improved the content of volatile fatty acids in the rumen (Deng et al., 2017). All the above research show that there are differences between soluble dietary fiber (SDF) and insoluble dietary fiber (IDF) in regulating microbial composition, but they all have certain regulating effects on the microbes of the offspring piglets.

Bamboo is one of the plants with the fastest growth rate, which is widely distributed all over the world. Bamboo has 1,575 species, with abundant resources (Basumatary et al., 2015). The fiber contents in powder processed from bamboo poles of three different varieties were all above 60%, which had a promising application prospect as a source of dietary fibers. The bamboo fiber was weakly acidic, with a low water solubility index (2.5–7.5%) (Felisberto et al., 2017). IDF of bamboo shoots had a porous surface, with much higher adsorption capacity than that of SDF. Compared with the control group, the total short-chain fatty acids of the group added with IDF and SDF were increased by 1.28 times and 0.71 times, respectively (Wu et al., 2020). Feeding bamboo powder is rich in fiber, which has the potential for beneficial dietary fiber. Our group found that the micronized bamboo powder processed from the yellow part of bamboo poles could reduce the number of *Escherichia coli* in the feces of weaned piglets and improve the growth performance (Dai et al., 2021). It could also improve the composition of the intestinal chyme flora of broilers and increase the average daily gain (Dai et al., 2022). This study further studied the effects of supplementary feeding of bamboo powder in the perinatal period of sows on fecal microorganisms of both sows and their offspring piglets. It may explore the practical value as a feeding supplement for sows.

## Materials and methods

### Ethics approval

All animal care and handling were approved by the Ethics Committee for Animal Experimentation, Scientific Research Department, Leshan Normal University, Sichuan, China.

### Feeding bamboo powder

The bamboo poles of *Phyllostachys pubescens* (from Sichuan Province, China) after growing for 5 ~ 6 years were collected, and the green parts of the poles were removed (Huang et al., 2019). The bamboo poles were first processed with a cutter grinder (Model 600, Zhengzhou Chuangyi Machinery Equipment Co., Ltd.), then dried until the water content was 10% ~ 12%. The bamboo powder was further ground with a hammer mill (Model 968, Jiangsu Muyang Group Co., Ltd.), and then passed through the 80-mesh sieve to obtain feeding bamboo powder. Regarding the method reported in previous literature, the water content (method 934.01; AOAC International, 2007), crude protein (method 990.03; AOAC International, 2007), and ash (method 942.15; AOAC International, 2007) were determined to be 8.45, 1.51, and 1.39%, respectively, and the neutral detergent fiber (NDF) and acid detergent fiber (ADF) (Van Soest et al., 1991) were determined to be 82.8 and 75.0%, respectively.

### Grouping and treatment

Thirty (30) healthy sows (Landrace × Yorkshire) with second parity and medium backfat thickness were selected. All sows were treated with estrus synchronization, artificially inseminated during the same period and fed with the same diet from mating to d 107 of gestation. Then, they were randomly divided into 3 groups, with 10 replicates in each group and 1 sow in each replicate. In the control group, the sows were fed a basal diet. In TRE1 group and TRE2 group, the sows were fed a basal diet and supplemented with 30 g d^−1^ and 60 g d^−1^ bamboo powder (24.84 g d^−1^ and 49.68 g d^−1^ NDF), respectively. The basal diet was prepared according to NRC2012 and SEGE 27th edition Sow nutrition standards. The composition and nutrition level of the diet was provided (Table 1).

**Table 1:** Composition and nutrition level of the basal diet (Air-dried).

Composition	Weight	Nutrition level^b^	Content
Corn	580	Digestive energy, MJ/kg	13.62
Flour	40	Net energy, MJ/kg	9.84
46%CP soybean meal	145	Crude protein, %	17.75
68%CP steamed fishmeal	20	Neutral detergent fiber, %	10.41
Fermented soybean meal	30	Acid detergent fiber, %	3.83
Extrudedsoybean	50	Digestible lysine, %	1.01
Soybean oil	10	Digestible lysine + methionine, %	0.56
Glucose	10	Digestible threonine, %	0.63
Wheat bran	55	Digestible tryptophan, %	0.18
Limestone	11	Digestible valine, %	0.71
Calcium hydrophosphate	12	Calcium, %	0.86
Salt	4	Total phosphorus, %	0.64
Sodium bicarbonate	1.5		
50% Choline chloride	1.5		
Premix^a^	30		
Total	1,000		

^a^Supplied the following per kilogram of diet: vitamin A, 12,000 IU; vitamin D3, 2,000 IU; vitamin E, 75 mg; vitamin K3, 4.5 mg; vitamin B1, 2.9 mg; vitamin B2, 10 mg; vitamin B6, 3.1 mg; vitamin B12, 0.04 mg; vitamin C, 100 mg; pantothenic acid, 26 mg; nicotinic acid, 31 mg; folic acid, 3.1 mg; choline, 500 mg; Fe, 120 mg; Cu, 15 mg; Zn, 70 mg; Mn, 65 mg; I, 0.4 mg; Co, 0.2 mg; Se, 0.3 mg; and Cr, 0.2 mg. ^b^Indicating that the nutritional level was the calculated value.

### Feeding and management

The experiment was conducted in the farm of Tianzhen Vica Agriculture and Animal Husbandry Food Co., Ltd., Datong, Shanxi Province of China. The experiment was conducted from the 107th day of gestation to the 7th day after delivery. On day 107 of gestation, sows were moved to individual farrowing pen with crates and fed two meals a day at 6:30 and 15:30, respectively. From the 107th day of gestation to delivery, 3 kg diet was fed every day. For the 7 days after delivery, the diet was fed with equal increment, of which 1 kg was given on the 1^st^ day after delivery, and 1 kg was added every day thereafter. During the experiment, all sows and piglets were allowed to drink freely. The sows were managed according to the normal feeding and health care procedures of farms.

### Sample collection

Serum samples of sows: On the morning of the 7th day after delivery, 5 sows with fasting for 12 h were randomly selected from each group. 5 ml of blood was drawn from the ear vein and put into the centrifuge tube, then left on ice for 30 min, centrifuged at 3000 rpm for 15 min, and then the serum could be separated. The collected serum was put into a 1.5 ml centrifuge tube and frozen at −20°C for testing.

Fecal samples: From the 5th to the 7th day after delivery, fresh fecal samples of all test sows were collected for three consecutive days, put into sample bags, and frozen at −20°C for detecting physicochemical indexes. On the morning of the 7th day after delivery, 5 sow fecal samples in each group were randomly selected, and about 300 mg of fecal samples were scraped and put into a 1.5 ml centrifuge tube. Merged fecal samples from every two sows into one sample. At the same time, about 300 mg of fresh feces from piglets of sows were collected and put into a 1.5 ml centrifuge tube, which were frozen with liquid nitrogen and frozen at −80°C for microbial analysis. Collected 2 piglet fecal samples from each litter, and merged every two replicates into one sample.

### Production performance of sows

The total number born and number born alive of each sow were recorded. The birth time of the first and the last piglets was accurately recorded, and the time interval between them was the farrowing duration. Mean of birth interval was calculated as: Farrowing duration/Total number born.

### Determination of serum biochemical indexes

The serum biochemical indexes of sows involved glucose, triglyceride, total cholesterol, total protein, urea nitrogen, superoxide dismutase, and malondialdehyde, all of which were determined by kits (Nanjing Jiancheng Institute of Bioengineering, Nanjing, China). The serum glucose, triglyceride, total cholesterol, and urea nitrogen were determined by enzymatic assays, with the kits of F006, A110, A111, and C013. The total serum protein was determined by ceruloplasmin assay, with the kit of A029. The serum superoxide dismutase was determined by hydroxylamine assay, with the kit of A001. The serum malondialdehyde was determined by the thio-barbituric acid method, with the kit of A003.

### Determination of physicochemical indexes of feces

The water content of feces was determined according to the previous report (Shang et al., 2021a). The fecal samples frozen at −20°C were thawed at room temperature. Two hundred gram of the samples were weighed and dried at 103°C for 72 h. The weight of samples before and after drying was determined, thus calculating the water content. The pH value of feces was determined according to the reference method with appropriate modification (Yang et al., 2010). 0.5 g of thawed feces was put into a beaker, then 20 ml of distilled water was added, stirred thoroughly, then left to stand, and the pH value was measured with a pH meter.

### Microbial flora analysis of feces

Genomic DNA was extracted from feces samples of sows and piglets by DNA extraction kit (Omega Bio-Tek, Norcross, GA, United States). The quality of the extracted DNA was detected by 1% agarose gel electrophoresis, and the DNA concentration and purity were determined by Nanodrop 2000 (Thermo scientific Inc., United States). With the above DNA as a template, the variable region of V3-V4 of 16S rRNA gene was amplified by PCR, with primers 338F (5′-ACTCCTACGGGAGGCAGCAG-3′) and 806R (5′-GGACTACHVGGGTWTCTAAT-3′) (Liu et al., 2016). The 16S rRNA PCR amplification products were Paired-end sequenced using Illumina Miseq PE300 platform by Majorbio (Shanghai, China)^1^. The raw reads have been deposited into NCBI with project NO. PRJNA953182.

Fastp^2^ (version 0.19.6) software was used to control the quality of the original sequence obtained with Paired-end sequencing (Chen et al., 2018). The FLASH^3^ (version 1.2.11) software was used for splicing (Magoč and Salzberg, 2011): (1) fastp (see footnote 2; version 0.19.6) was applied to control the quality of the original sequence of Paired-end sequencing (Chen et al., 2018). FLASH (see foot note 3) (Version 1.2.11) was applied for splicing (Magoč and Salzberg, 2011): (1) Filtering the bases with mass value below 20 at the end of the reads, and setting a window at 50 bp. If the average mass value in the window was below 20, removing the back bases from the window, filtering the reads with the mass value below 50 bp after quality control, and removing the reads with N bases. (2) Merging the paired reads into one sequence according to the overlap relationship between PE reads, with a minimum overlap length of 10 bp. (3) Screening non-conforming sequences. The maximum allowable mismatch ratio in the overlap region of merged sequences was set as 0.2. (4) Differentiating samples according to barcodes and primers at the beginning and end of the sequence, and adjusting the sequence direction. The allowable mismatch number of barcodes was set as 0, and the maximum primer mismatch number was set as 2. UPARSE^4^ (version 7.1) (Stackebrandt and Goebel, 1994; Edgar, 2013) was applied for Operational taxonomic unit (OTU) clustering of the sequences spliced by quality control according to the similarity of 97%, and chimeras were removed. To minimize the effects of sequencing depth on the subsequent analysis of Alpha diversity and Beta diversity data, the sequence number of all samples was flattened to 34,060, and the Good’s coverage of each sample could still reach 99.49% after flattening. RDP classifier^5^ (version 2.11) was applied to align the Silva 16S rRNA gene database (Release 138^6^) for conducting OTU species taxonomic annotation (Wang et al., 2007), and the confidence threshold was 70%. The community composition of each sample was counted at different specie classification levels.

### Data processing and statistical analysis

SPSS23.0 was applied for one-way ANOVA, and LSD method was used for multiple comparisons. The statistical analysis of production performance, serum biochemical indexes, and fecal physicochemical indexes were performed, and the results were expressed by Mean ± Standard deviation, *p* < 0.05 indicated a significant difference, and 0.05 ≤ *p* < 0.10 indicated a difference trend.

The 16S rRNA sequencing data of all samples were analyzed with Majorbio Cloud platform,^7^ as follows: Mothur^8^ was used to calculate the Alpha diversity, including Ace, Chao, and Sobs indices to represent richness, Shannon and Simpson indices to represent diversity. Wilxocon rank sum test was used to analyze the difference of Alpha diversity between groups.

Principal coordinate analysis (PCoA analysis) based on Bray–Curtis distance algorithm was used to test the similarity of microbial community structure among samples, and ANOSIM nonparametric test was used to analyze whether the difference of microbial community structure among sample groups was significant. Student’s T test was used to carry out two-tailed test for analyzing the difference of relative abundance of main microorganisms in different experimental groups at phylum and genus levels. *p* < 0.05 indicated a significant difference, and 0.05 ≤ *p* < 0.10 indicated a difference trend.

## Results

### Effects of feeding bamboo powder on the farrowing duration of sows

From the data in Table 2, it could be observed that the total number born, number born alive, farrowing duration, birth interval of TRE1 and TRE2 groups were not significantly different from that of the control group (*p* > 0.05). Supplementary feeding of 30 g d^−1^ or 60 g d^−1^ bamboo powder had no significant effect on the farrowing duration of sows (*p* > 0.05). The farrowing duration and mean of birth interval of sows were relatively low for sows in the group supplemented with 60 g d^−1^ bamboo powder.

**Table 2:** Effects of feeding bamboo powder on the farrow duration of sows.

Items	Control	TRE1	TRE2	*p-*value
Total number born	10.75 ± 2.44	12.00 ± 1.58	11.25 ± 1.75	0.563
Number born alive	10.50 ± 2.00	11.60 ± 2.07	11.25 ± 1.75	0.573
Farrowing duration/min	271.25 ± 60.81	316.00 ± 62.69	262.50 ± 32.84	0.204
Mean of birth interval/min	25.63 ± 4.47	26.28 ± 3.60	23.74 ± 4.27	0.521

In the same row, values with no letter means no significant difference between the two groups (*p* < 0.05).

### Effects of feeding bamboo powder on serum biochemical indexes of sows

From the data in Table 3, it could be observed that, the levels of total cholesterol, triglyceride, and malondialdehyde (MDA) in the serum of sows decreased with the increased amount of feeding bamboo powder. The levels of total cholesterol and triglyceride in TRE1 and TRE2 groups were significantly lower than those in the control group (*p* < 0.05). Compared with the control group, the MDA level in the serum of sows decreased by 82.15 and 71.27%, respectively, both with significant differences (*p* < 0.05). Supplementary feeding 30 g d^−1^ or 60 g d^−1^ bamboo powder had no significant effect on levels of glucose, total protein, urea nitrogen, and superoxide dismutase activity in the serum of sows (*p* > 0.05).

**Table 3:** Effects of feeding bamboo powder on the serum biochemical indexes of sows.

Items	Control	TRE1	TRE2
Glucose/mmol L^−1^	0.40 ± 0.10	0.67 ± 0.28	0.29 ± 0.06
Triglyceride/mmol L^−1^	0.68 ± 0.11^a^	0.59 ± 0.06^ab^	0.39 ± 0.05^b^
Total cholesterol/mmol L^−1^	3.42 ± 0.27^a^	3.36 ± 0.30^a^	2.25 ± 0.20^b^
Total protein/mg mL^–1^	81.86 ± 2.22	76.15 ± 5.27	81.04 ± 2.55
Urea nitrogen/mmol L^−1^	19.90 ± 0.66	20.81 ± 0.81	21.21 ± 0.46
Superoxide dismutase/U mL^−1^	24.59 ± 1.52	25.71 ± 1.09	23.34 ± 1.10
Malondialdehyde /nmol mL^−1^	27.67 ± 9.11^a^	7.95 ± 1.45^b^	4.94 ± 0.93^b^

In the same row, values with no letter or same letter superscripts mean no significant difference between the two groups (*p* > 0.05), while with different letter superscripts mean significant difference between the two groups (*p* < 0.05).

### Effects of feeding bamboo powder on the physicochemical indexes of sow feces

From the data in Table 4, the pH values of sow feces in TRE1 and TRE2 groups were significantly higher than those in control group (*p* < 0.05). The pH value of feces was not significantly different between the two treatment groups (*p* > 0.05). The fecal moisture content of sows in the TRE1 and TRE2 groups was increased by 8.20% (*p* > 0.05) and 12.99% (*p* < 0.05), respectively. Compared with the control group, there was no significant difference in fecal moisture between TRE1 and TRE2 groups (*p* > 0.05).

**Table 4:** Effects of feeding bamboo powder on the physicochemical indexes of feces of sows.

Items	Control	TRE1	TRE2
pH	6.09 ± 0.08^a^	6.32 ± 0.02^b^	6.35 ± 0.08^b^
Water content/%	65.45 ± 5.27^a^	70.82 ± 4.85^ab^	73.95 ± 1.09^b^

In the same row, values with same letter superscripts mean no significant difference between the two groups (*p* > 0.05), while with different letter superscripts mean significant difference between the two groups (*p* < 0.05).

### Effects of feeding bamboo powder on the microbial sequence data and diversity of alpha and beta in feces of sows and offspring piglets

**Figure 1:**
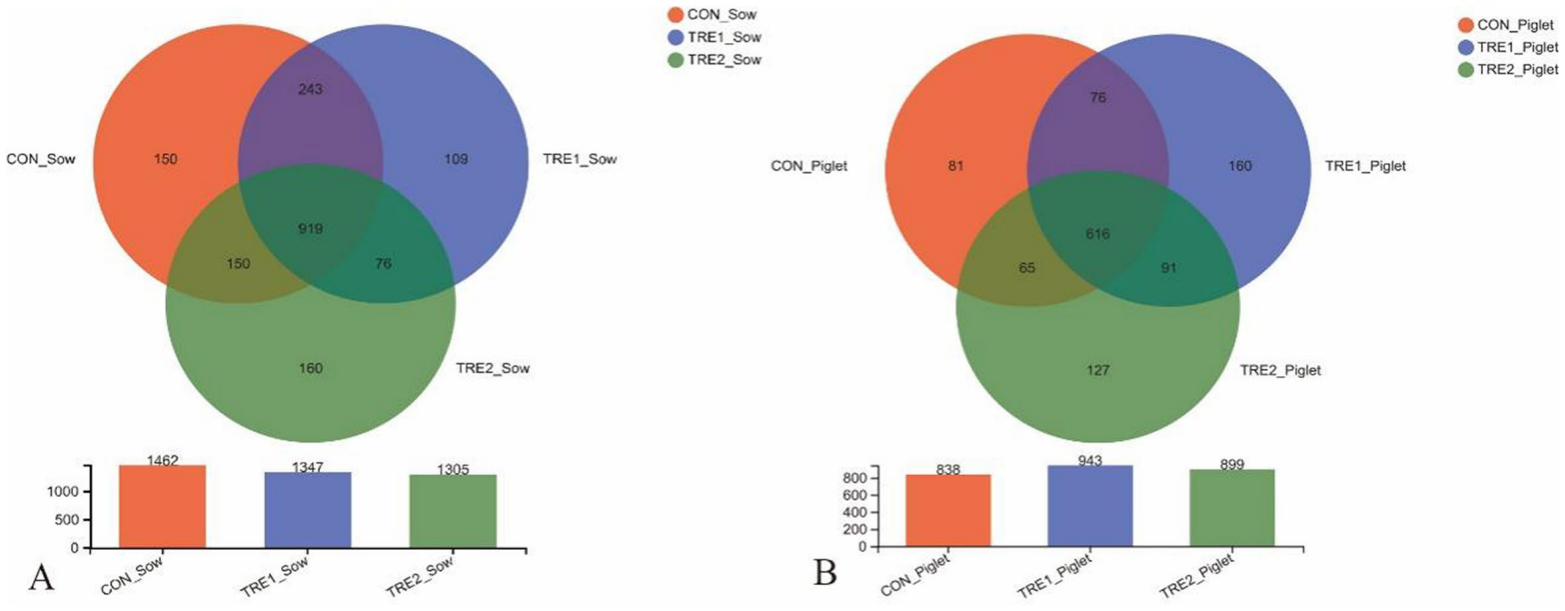
The Venn diagram for OTUs of the fecal microbiota of sows and offspring piglets in the control group, TRE1 group, and TRE2 group.

The effects of feeding bamboo powder on the diversity and richness of microorganisms in the feces of sows and offspring piglets were explored. For each sample, the bacterial 16S rRNA V3-V4 region was amplified by PCR and analyzed with Illumina Miseq high-throughput sequencing. According to the statistics of the flattened OTUs (Figure 1), there were 1,462 OTUs in the feces of sows in the control group, and the OTUs in the feces of sows were 1,347 and 1,305 in TRE1 and TRE2 groups, which were lower than those of the control group. The unique OTUs were 185 and 236 in TRE1 and TRE2 groups, indicating that feeding bamboo powder in the perinatal period might regulate the microorganism in the feces of sows.

For the offspring piglets, there were 838 OTUs in the feces of the control group, and the OTUs were 943 and 899 in TRE1 and TRE2 groups, which were higher than that of the control group. The unique OTUs were 251 and 218 in TRE1 and TRE2 groups, indicating that feeding bamboo powder could regulate the fecal microbial composition not only in sows, but also in their offspring piglets.

From the data in Table 5, it could be observed that the richness indexes (including Ace, Chao, and Sobs) in groups supplemented with feeding bamboo powder were lower than those in the control group. The Chao index of TRE2 group was significantly lower than that of the control group (*p* < 0.05), and TRE1 group tended to be lower than that of the control group (*p* < 0.10). Both the Ace and Sobs indexes of TRE2 group tended to be lower than those of the control group (*p* < 0.10).

**Table 5:** Effects of feeding bamboo powder on alpha diversity of fecal microbiota for sows and piglets.

Items		Control	TRE1	TRE2
Sow	Ace	1158.10 ± 111.12	997.09 ± 166.49	971.09 ± 184.93
	Chao	1162.50 ± 88.69^a^	998.05 ± 164.31^ab^	903.87 ± 192.3^b^
	Sobs	857.60 ± 116.57	737.2 ± 147.72	675.80 ± 177.05
	Shannon	4.21 ± 0.32	3.85 ± 0.19	3.76 ± 1.01
	Simpson	0.0496 ± 0.0209	0.0625 ± 0.0171	0.0998 ± 0.0956
Piglet	Ace	563.15 ± 108.12	706.98 ± 103.76	641.91 ± 84.19
	Chao	574.99 ± 106.19	685.67 ± 66.94	628.58 ± 92.68
	Sobs	442.2 ± 83.77	504.85 ± 62.94	468.00 ± 67.85
	Shannon	3.67 ± 0.18	3.53 ± 0.29	3.41 ± 0.42
	Simpson	0.0750 ± 0.0282	0.0961 ± 0.0268	0.1139 ± 0.0671

In the same row, values with no letter or same letter superscripts mean no significant difference between the two groups (*p* > 0.05), while with different letter superscripts mean significant difference between the two groups (*p* < 0.05).

The results were different from the offspring piglets. The richness indexes (including Ace, Chao, and Sobs) in TRE1 and TRE2 groups were higher than those of the control group. Ace and Chao indexes in TRE1 group tended to be higher than those of the control group (*p* < 0.10). The diversity indexes (Shannon and Simpson) of the fecal microbial flora of both sows and piglets in TRE1 and TRE2 groups were not significantly different from those of control group (*p* > 0.05).

**Figure 2:**
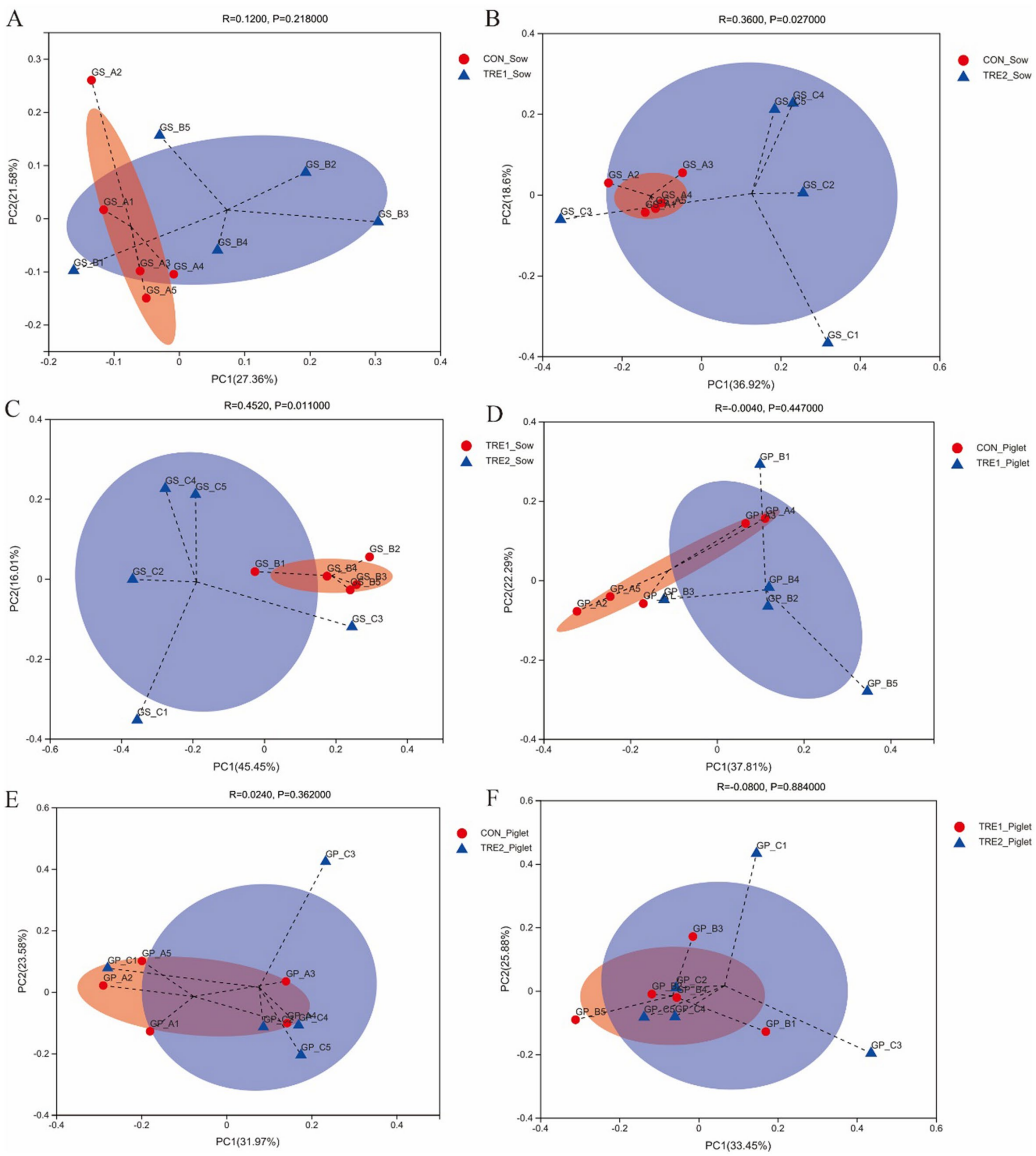
Beta-diversity analysis among experimental groups. PCoA for sow between the control group (CON_Sow) and TRE1 group (TRE1_Sow) **(A)** and TRE2 group (TRE2_Sow) **(B)**, and between TRE1 (TRE1_Sow) and TRE2 (TRE2_Sow) groups **(C)**. PCoA for piglets between the control group (CON_Piglet) and TRE1 (TRE1_Piglet) **(D)** and TRE2 (TRE2_Piglet) groups **(E)**, and between TRE1 (TRE1_Piglet) and TRE2 (TRE2_Piglet) groups **(F)**. *N* = 5.

For exploring the effects of feeding bamboo powder on beta diversity of fecal microbiota, principal coordinate analysis (PCoA) was used to determine the difference between groups. The PCoA was conducted based on the Braye–Curtis distance of OTU relative abundance in the fecal microbiota of sows and piglets. As could be seen from Figure 2A, the sow fecal microbiota was similar between TRE1 and control groups (ANOSIM: *R* = 0.1200, *p* = 0.218). From Figures 2B,C, we could know the fecal microbiota of TRE2 and control groups were separated (ANOSIM: *R* = 0.3600, *p* = 0.027), and TRE1 and control groups were also separated (ANOSIM: *R* = 0.4520, *p* = 0.011). According to Figures 2D–F, the piglet fecal microbiota of TRE1 and control groups were not separated (ANOSIM: *R* = 0.0040, *p* = 0.447). Similarly, TRE2 and control groups (ANOSIM: *R* = 0.0240, *p* = 0.362), TRE2 and TRE1 groups (ANOSIM: *R* = 0.0800, *p* = 0.884).

### Effects of feeding bamboo powder on microbial flora species in feces of sows and piglets

**Figure 3:**
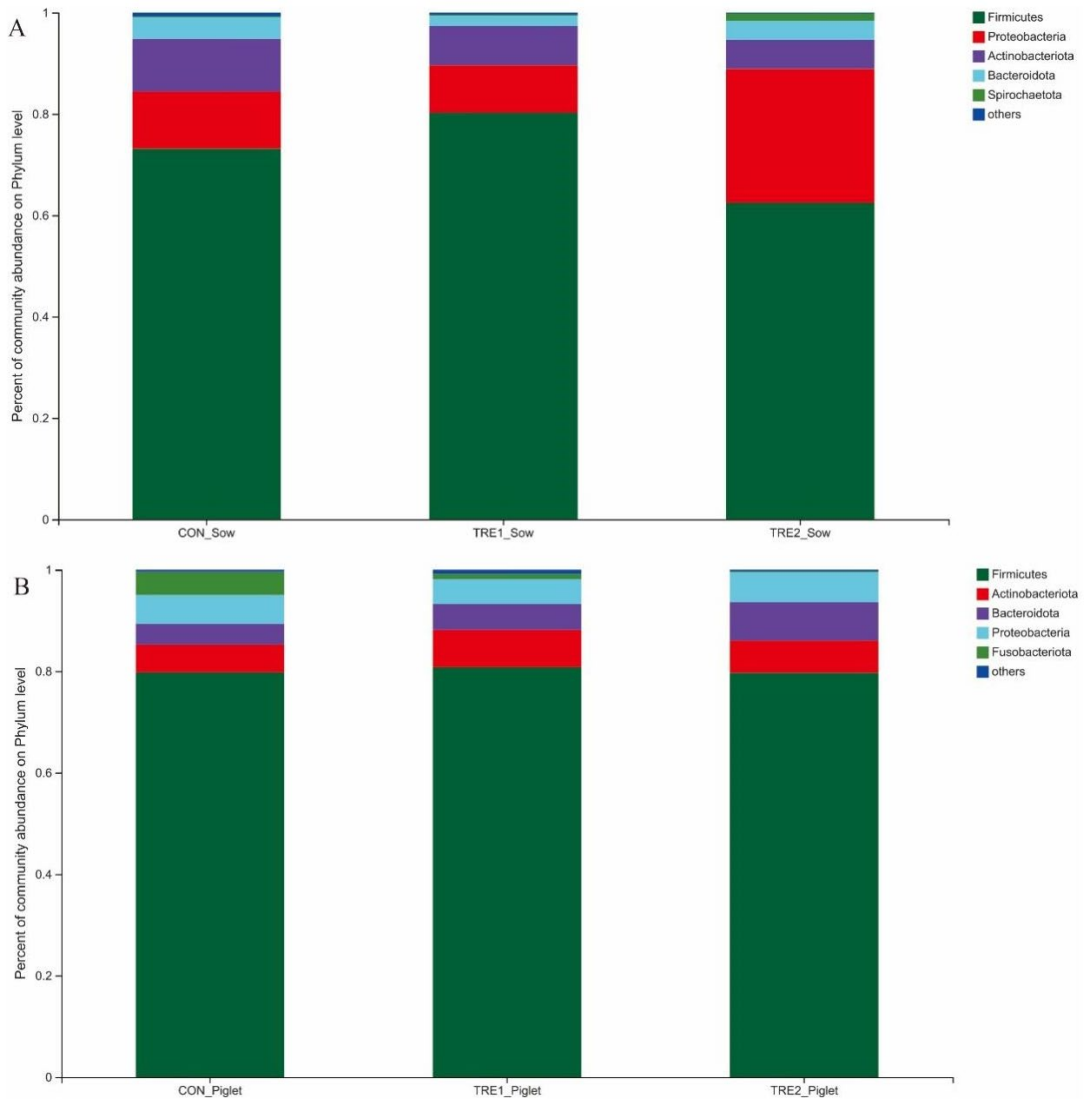
Fecal microbiota species of sows and piglets at phylum level among different groups. **(A)** Fecal microbiota for sows among different groups. Sows are regarded as the experimental units, *n*= 5 for each group. **(B)** Fecal microbiota for piglets among different groups. Piglets are regarded as the experimental units, *n*= 5 for each group.

The microflora species in the feces of sows and piglets in different groups at the phylum level were shown in Figure 3. From Figure 3A, the first five dominant bacteria shared in three groups were *Firmicutes*, *Proteobacteria*, *Actinobacteriota*, *Bacteroidota*, and *Spirochaetota.* In TRE1 group, the proportions of *Firmicutes* (80.25% vs. 73.15%) and *Spirochaetota* (0.21% vs. 0.19%) were increased compared with the control group, while the proportions of *Proteobacteria* (9.35% vs. 11.29%), *Actinobacteriota* (7.71% vs. 10.37%), and *Bacteroidota* (2.06% vs. 4.27%) were decreased. In TRE2 group, the proportions of *Proteobacteria* (26.43% vs. 11.29%) and *Spirochaetota* (1.39% vs. 0.19%) were increased compared with the control group, while the proportions of *Firmicutes* (62.46% vs.73.15%), *Actinobacteriota* (5.74% vs. 10.37%), and *Bacteroidota* (3.76% vs. 4.27%) were decreased. In contrast with the control group, the proportions of *Firmicutes* and *Spirochaetota* changed greatly in TRE2 group, which were decreased by 10.69% and increased by 15.14%, respectively.

From Figure 3B, there was a new dominant specie in the fecal microflora of piglets over sows, which was *Fusobacteriota*. In TRE1 group, the proportions of *Firmicutes* (80.77% vs. 79.74%), *Actinobacteriota* (7.37% vs. 5.53%), and *Bacteroidota* (5.10% vs. 4.09%) were increased compared with the control group, while the proportions of *Fusobacteriota* (1.01% vs. 4.58%) and *Proteobacteria* (4.90% vs. 5.68%) were decreased. In TRE2 group, the proportions of *Actinobacteriota* (6.35% vs. 5.53%), *Bacteroidota* (7.58% vs. 4.09%), and *Proteobacteria* (5.93% vs. 5.68%) were increased compared with the control group, while proportions of *Fusobacteriota* (0.22% vs. 4.58%) and *Firmicutes* (79.68% vs. 79.74%) were decreased. In contrast with the control group, the proportions of *Fusobacteriota* changed greatly in both TRE1 and TRE2 groups, which were decreased by 3.57 and 4.36%, respectively.

**Figure 4:**
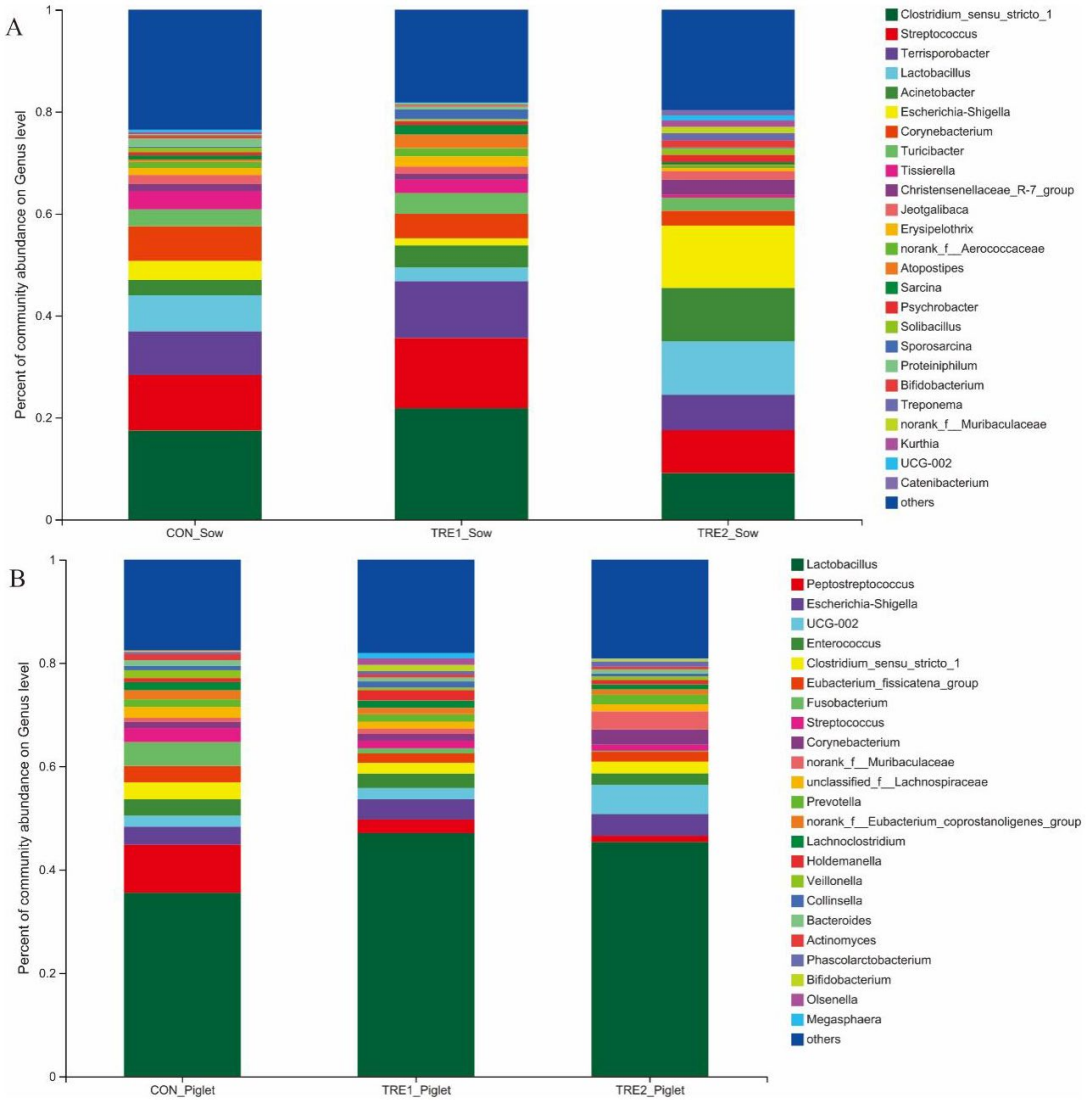
Fecal microbiota species of sows and piglets at genus level among different groups. **(A)** Fecal microbiota for sows among different groups. Sows are regarded as the experimental units, N= 5 for each group. **(B)** Fecal microbiota for piglets among different groups. Piglets are regarded as the experimental units, N= 5 for each group..

The microflora species in the feces of sows and piglets in different groups at the genus level were shown in Figure 4. From Figure 4A, there were 25 dominant bacteria at the genus level in the feces of sows of all three groups, including *Clostridium_sensu_stricto_*1, *Streptococcus*, *Terrisporobacter*, *Lactobacillus*, *Acinetobacter*, *Escherichia-Shigella*, *Corynebacterium*, *Turicibacter*, *Tissierella*, *Christensenellaceae_R-7*_group, *Jeotgalibaca*, *Erysipelothrix*, norank*_f_Aerococcaceae*, *Atopostipes*, *Sarcina*, *Psychrobacter*, *Solibacillus*, *Sporosarcina*, *Proteiniphilum*, *Bifidobacterium*, *Treponema*, norank_*f_Muribaculaceae*, *Kurthia*, *Ruminococcaceae UCG-002*, and *Catenibacterium*. The proportions of all dominant bacteria were 76.49, 81.81, and 80.30% in the control group, TRE1 group, and TRE2 group, respectively.

From Figure 4B, there were 24 dominant bacteria in the feces of piglets of all three groups. The proportions of all dominant bacteria were 82.49, 81.94, and 80.88% in the control group, TRE1 group, and TRE2 group, respectively. Different from the dominant bacterial species in the feces of sows, 17 unique dominant bacteria were observed in piglets, including *Peptostreptococcus*, *Enterococcus*, *Eubacterium_fissicatena_group*, *Fusobaterium*, *Streptococcus*, unclassified*_f_Lachnospiraceae*, *Prevotella*, Norank_*f_Eubacterium_coprostanoligenes_group*, *Lachnoclostrium*, *Holdemanella*, *Veillonella*, *Collinsella*, *Bacteroides*, *Actinomyces*, *Phascolarctobacterium*, *Olsenella*, and *Megasphaera.*

**Figure 5:**
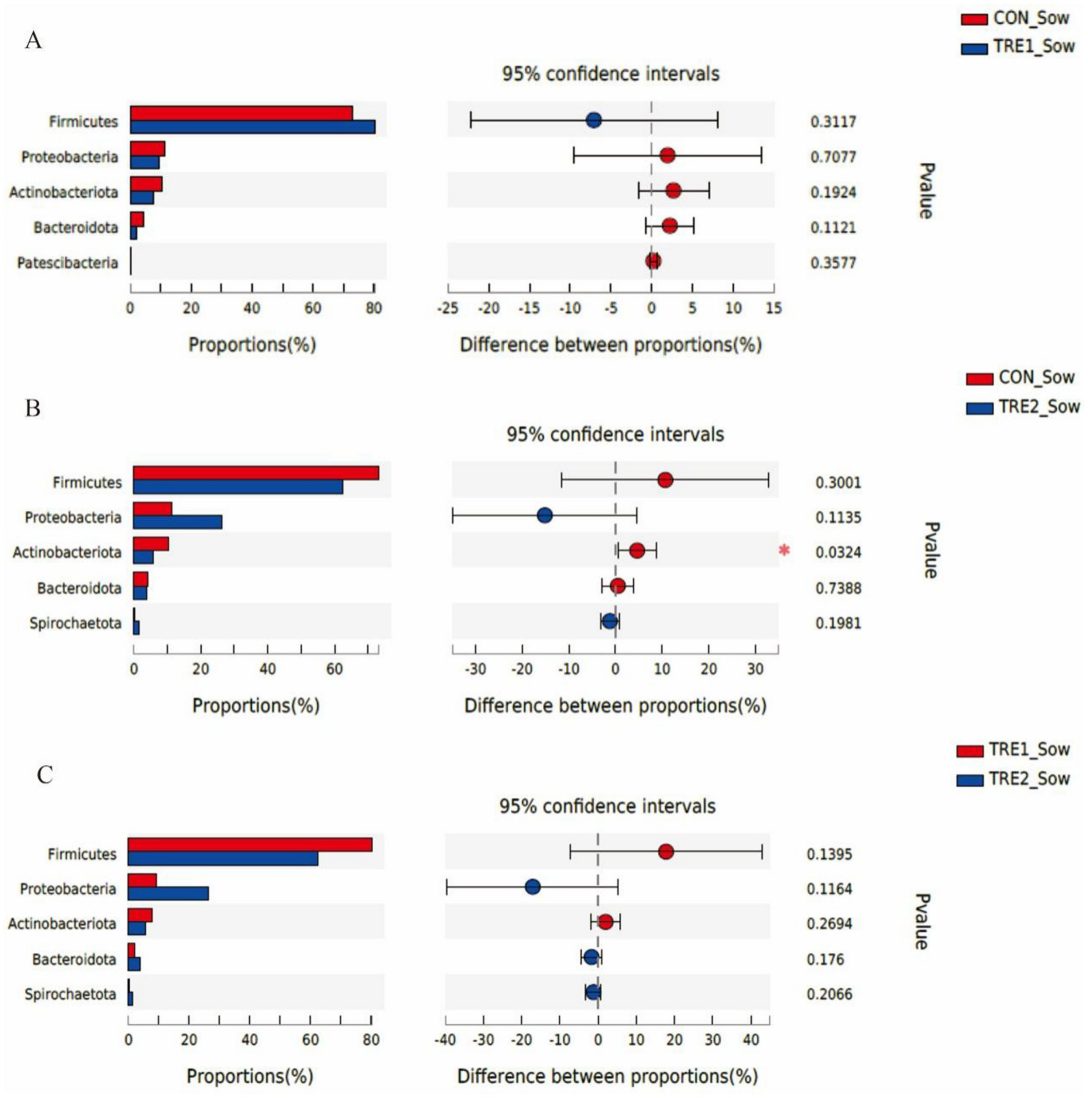
Differences in fecal microbiota of sows at phylum level among different groups. **(A)** The difference of fecal microbiota for sows between the control group and TRE1 group. **(B)** The difference of fecal microbiota for sows between the control group and TRE2 group. **(C)** The difference of fecal microbiota for sows between TRE1 and TRE2 groups. Sows are regarded as the experimental units, *N* = 5 for each group.

**Figure 6:**
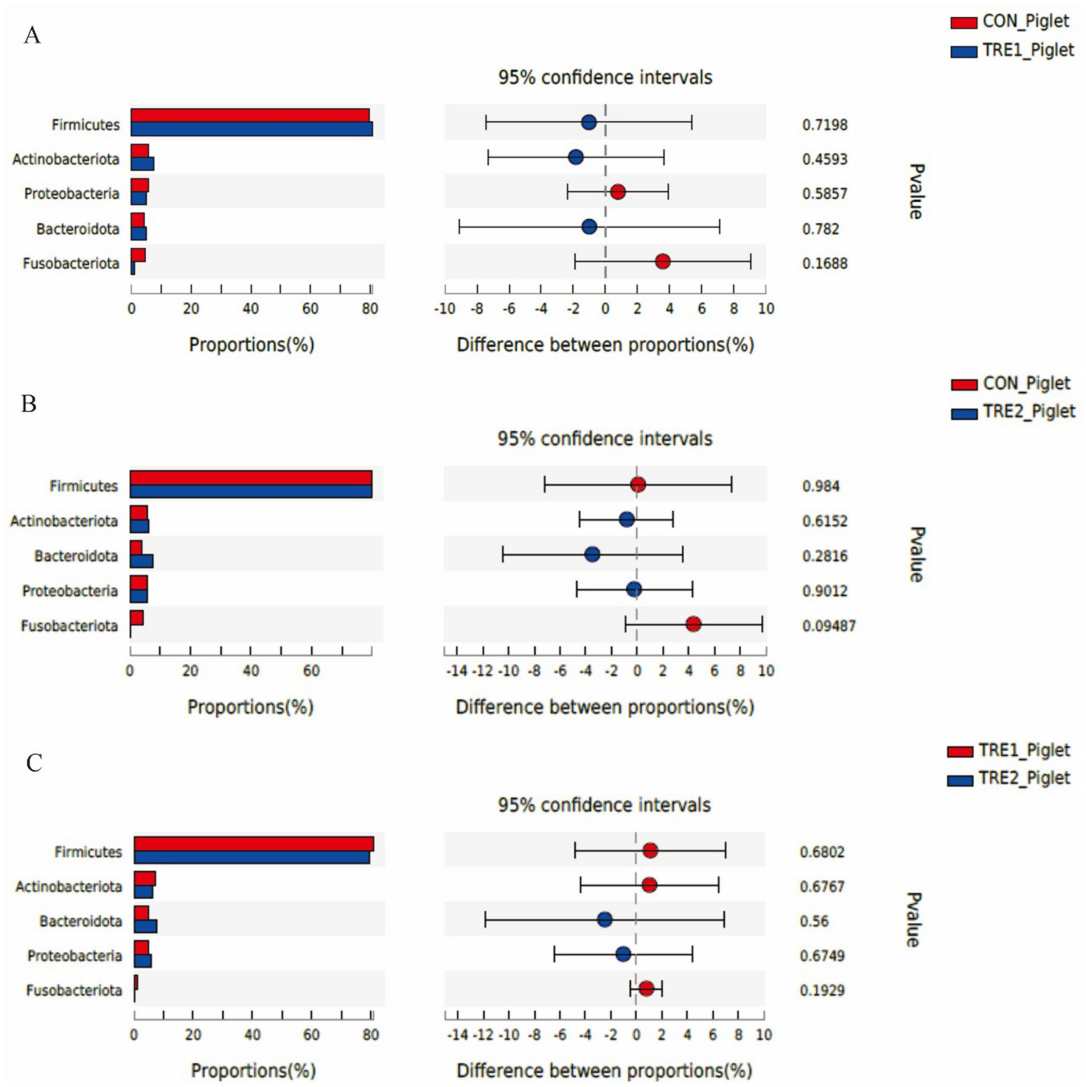
Differences in fecal microbiota of piglets at phylum level among different groups. **(A)** The difference in fecal microbiota for piglets between control group and TRE1 group. **(B)** The difference in fecal microbiota for piglets between control group and TRE2 group. **(C)** The difference in fecal microbiota for piglets between TRE1 and TRE2 groups. Piglets are regarded as the experimental units, *N* = 5 for each group.

The differences of fecal microflora species in sows and piglets at the phylum level were compared and analyzed in three groups (Figures 5, 6). The relative abundance of *Actinobacteriota* in the feces of sows in TRE2 group was significantly lower than that in control group (*p* < 0.05), but there was no significant difference in the bacterial species at the phylum level between the two treatment groups (*p* > 0.05) (Figure 5B). The fecal bacterial species at the phylum level were not significantly different between TRE1 and control groups, TRE1 and TRE2 groups (*p* > 0.05) (Figures 5A,C). The relative abundance of *Fusobacteriota* in the feces of piglets in TRE2 group tended to be lower than that in control group (*p* < 0.10), but there was no significant difference in the bacterial species between the two treatment groups (*p* > 0.05) (Figure 6B). The fecal bacterial species were not significantly different between piglets in TRE1 and control groups, TRE1 and TRE2 groups (*p* > 0.05) (Figures 6A,C).

**Figure 7:**
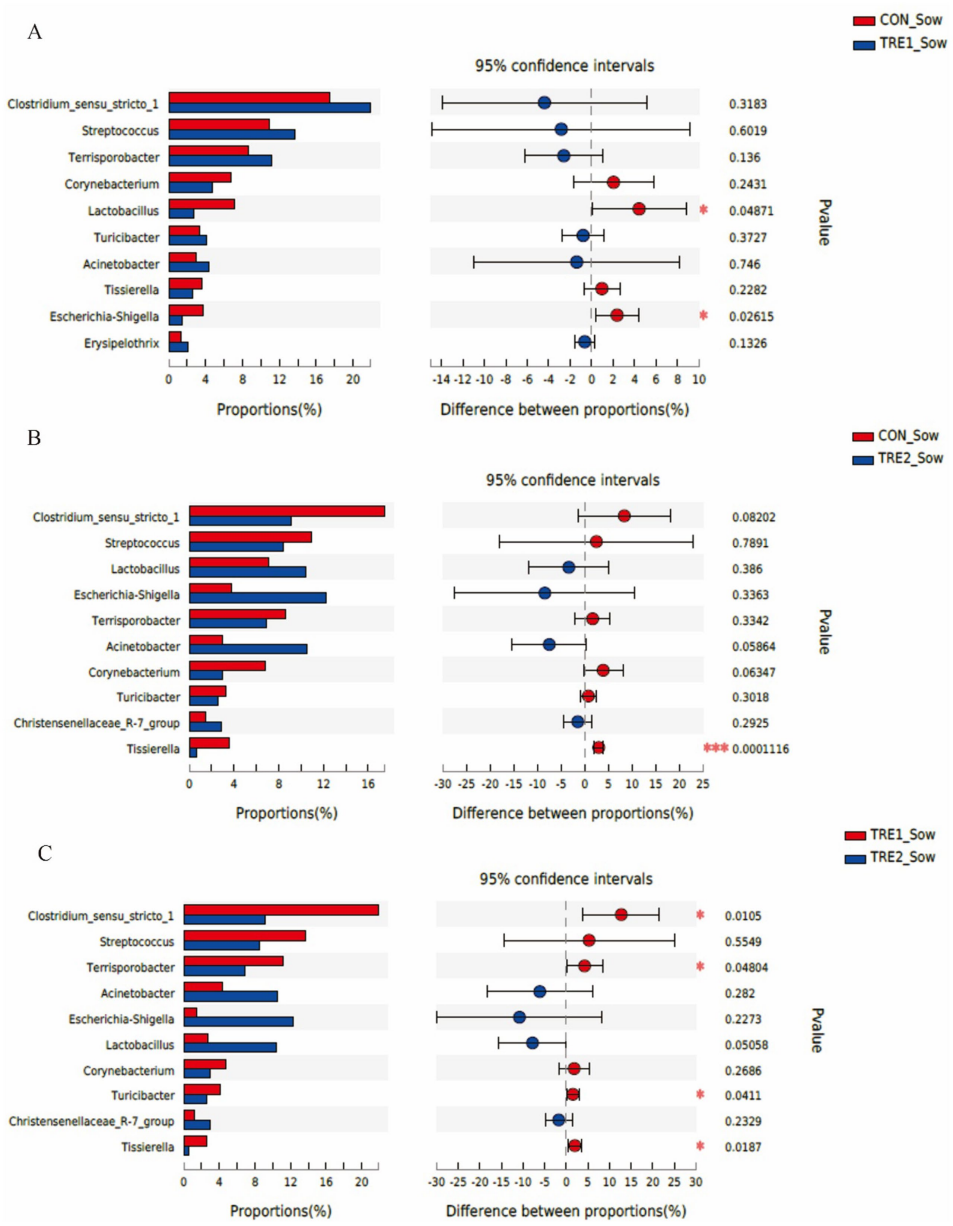
Differences in fecal microbiota of sows at genus level among different groups. **(A)** The difference in fecal microbiota for sows between the control group and TRE1 group. **(B)** The difference in fecal microbiota for sows between the control group and TRE2 group. **(C)** The difference of fecal microbiota for sows between TRE1 and TRE2 groups. Sows are regarded as the experimental units, *N* = 5 for each group.

**Figure 8:**
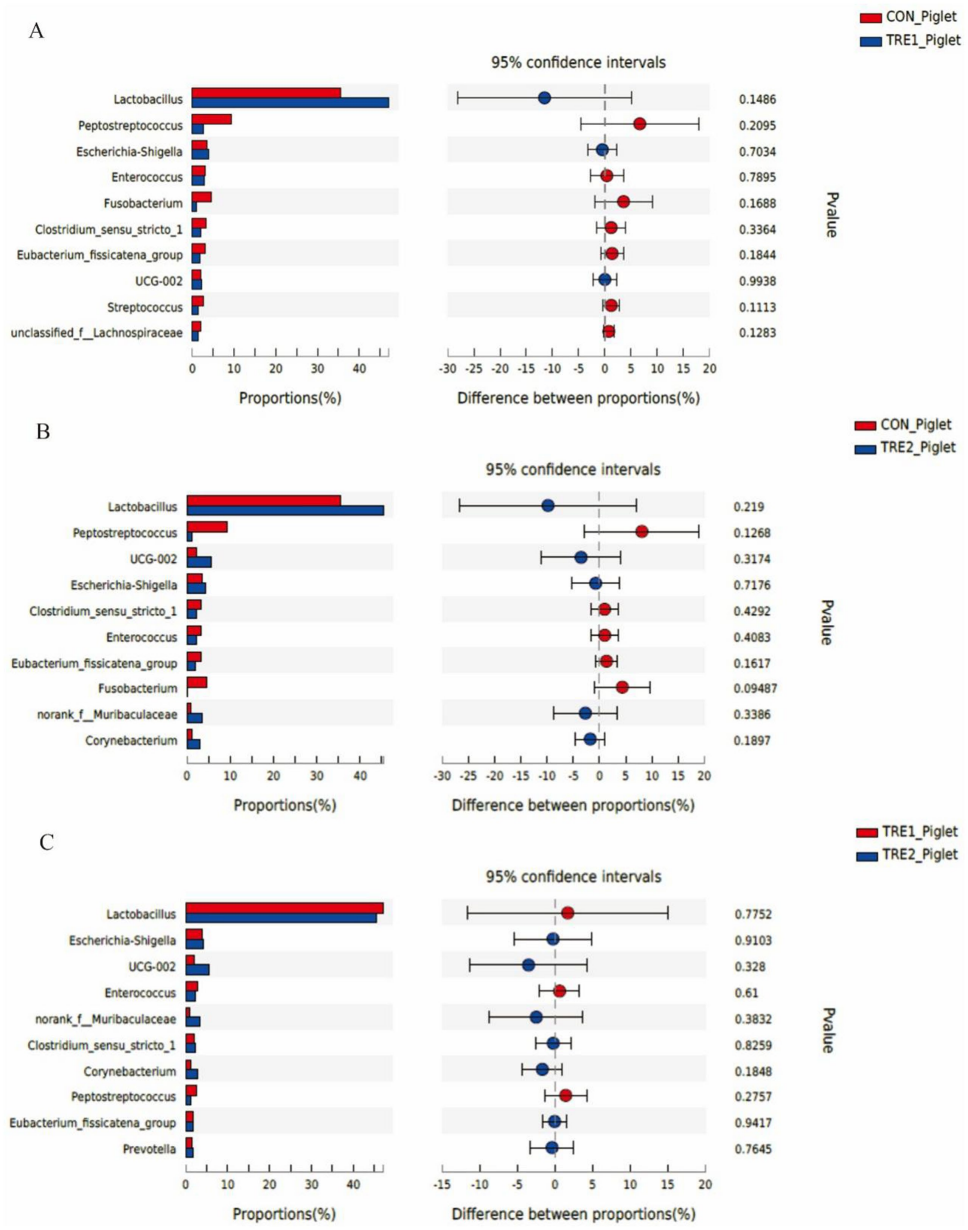
Differences in fecal microbiota of piglets at genus level among different groups. **(A)** The difference in fecal microbiota for piglets between the control group and TRE1 group. **(B)** The difference in fecal microbiota for piglets between the control group and TRE2 group. **(C)** The difference in fecal microbiota for piglets between TRE1 and TRE2 groups. Piglets are regarded as the experimental units, *N* = 5 for each group.

The top 10 dominant bacteria in the feces of sows and piglets at the genus level were compared and analyzed in three groups (Figures 7, 8). In TRE1 group, the relative abundance of *Lactobacillus* and *Escherichia-Shigella* in the feces of sows was significantly lower than that in control group (*p* < 0.05) (Figure 7A). In TRE2 group, the relative abundance of *Tissierella* in the feces of sows was significantly lower than that of the control group (*p* < 0.05), *Corynebacterium* tended to be lower than that of the control (*p* < 0.10), while *Acinetobacter* tended to be higher than that of the control (*p* < 0.10) (Figure 7B). In Figure 7C, the relative abundance of *Clostridium_sensu_stricto_*1, *Terrisporobacter*, *Turicibacter*, and *Tissierella* in the feces of sows in TRE2 group was significantly lower than that of TRE1 group (*p* < 0.05), while *Lactobacillus* tended to be higher than that of TRE1 group (*p* < 0.10). Among the top 10 dominant bacteria, the relative abundance of *Fusobacterium* in the feces of piglets in TRE2 group tended to be lower than that in control group (*p* < 0.10), but there was no significant difference in the bacterial species at the genus level between the two treatment groups (*p* > 0.05) (Figure 8B). The fecal bacterial species at the genus level were not significantly different between piglets in TRE1 and control groups, TRE1 and TRE2 groups (*p* > 0.05) (Figures 8A,C).

## Discussion

### Effects of feeding bamboo powder on the farrowing duration of sows

Many risk factors may affect the stillbirth rate of piglets on farms. The increased stillbirth rate was associated with certain production performances of sows, such as cumulative farrowing duration >90 min and birth interval > 30 min (Lanh and Nam, 2022). Thus, careful supervision of parturition might be good for reducing the stillbirth rate. A previous study found that the improvement of parturition duration by increasing fiber intake (33.5% vs. 17.5% NDF) during late gestation was associated with gut microbiota, production of short-chain fatty acids and other metabolites, potentially serving for energy metabolism (Liu et al., 2022). Under heat stress, a high-fiber diet (6.5% acid detergent fiber, ADF) reduced the expression of heat shock protein 70 in sows, showing the lower respiratory rate and shorter farrowing duration (Oh et al., 2022). Our study assumed that, supplementary feeding of bamboo powder in the perinatal period of sows might increase the fiber intake, thus improving the farrowing duration. However, it was found that feeding bamboo powder had no significant effect on the farrowing process and mean birth interval of sows, which might be related to the level of supplemented fibers. In this study, supplementary feeding 60 g d^−1^ bamboo powder was equivalent to increasing the intake of 49 g of NDF and 45 g of ADF per day. When the daily intake of feed was 3 kg, the calculated fiber level of TRE2 group in this study (12.04% NDF, 4.81% ADF) was still lower than that reported in the above two references.

It was assumed that different fiber materials could affect the farrowing process by regulating gut microbiota and energy metabolism. However, it was found that extra energy supplement did not improve the farrowing performance, and farrowing duration and stillbirth rate were not affected by dietary fiber (DF) sources in a trial with four DF sources for late gestating sows (Feyera et al., 2021). Another study showed that maternal dietary fiber intake from gestation d80 prevented the prolonged farrowing duration of sows and shortened the average piglet birth interval (Li et al., 2021). According to the above research, it was speculated that the fiber component of the feeding bamboo powder in this study might not be the main factor affecting the farrowing performance. The effects of bamboo powder on farrowing duration and stillbirth could be further evaluated by increasing the supplementation level and extending feeding period.

### Effects of feeding bamboo powder on serum and fecal biochemical indexes of sows

Fat metabolism in serum could be regulated by dietary fiber intake. It was found that supplementation of sugar beet pulp and wheat bran both reduced serum concentration of total cholesterol of sows (Shang et al., 2021a), and addition of flaxseed meal and oat hulls reduced fat digestibility and serum cholesterol of growing pigs (Ndou et al., 2017). Similarly, it was found that supplementary feeding of bamboo powder in the perinatal period of sows could also significantly reduced serum triglyceride and total cholesterol in this study. In the above studies, beet pulp and flaxseed meal mainly included soluble fiber, while bran, oat hulls, and feeding bamboo powder mainly included insoluble fiber, indicating that serum total cholesterol and triglyceride levels might be associated with dietary fiber level instead of fiber sources. Another study showed that the effects of fiber type on serum biochemical parameters for lipid metabolism were different at different stages of sows (Weng, 2020). Thus, it was necessary to explore the regulation effect of feeding bamboo powder in different physiological stages, and its effects on fat digestibility and milk fat content in sows.

Serum urea nitrogen could reflect the utilization efficiency of protein in animals (Kohn et al., 2005). The concentration of serum urea nitrogen might have been the result of dietary and/or body protein breakdown (Muller et al., 2022). It was showed that supplementation of sugar beet pulp instead of wheat bran could significantly reduce serum urea nitrogen of pregnant sows (Shang et al., 2021a). Another study found that serum total protein concentration was increased with increased inclusion level of fiber, and impaired growth performance in finishing pigs (Bakare et al., 2016). Our study found that feeding bamboo powder had no significant effect on serum total protein, urea nitrogen, and glucose concentration, indicating that feeding 60 g d^−1^ feeding bamboo powder in the perinatal period had no negative effect on metabolism and utilization of protein and sugar for sows.

Serum superoxide dismutase was an important biochemical index reflecting the redox status of the body (Ren et al., 2012), while serum malondialdehyde was often used to monitor the degree of lipid peroxidation (Li et al., 2016). The fiber composition in pregnancy diet played an important role in improving antioxidant capacity and reducing the inflammatory response of sows and their offspring through modulating the composition of gut microbiota (Li et al., 2019). It was showed that supplementation inulin rich in soluble fiber could improve the activity of superoxide dismutase, and reduce the concentration of malondialdehyde and proinflammatory cytokines in serum of dairy cows (Wang et al., 2021). Similarly, another previous study also found that dietary fermentable fiber could significantly improve lipid profile and oxidative status in the serum of patients, in which malondialdehyde level was significantly decreased (Xie et al., 2015). Our study found that supplementary feeding of bamboo powder could significantly reduce serum malondialdehyde of sows. It was found that supplementation of insoluble fiber or soluble fiber isolated from barley could both reduce serum malondialdehyde, while barley soluble fiber could improve butyric acid and insulin sensitivity (Li et al., 2020). Thus, it was necessary to explore whether feeding bamboo powder needs to be mixed with soluble fiber to improve fat acid and sugar metabolism in sows during the perinatal period.

The effects of supplementing fiber resources on the physicochemical indexes of sow feces may be related to the fiber characteristics. Adding fiber resource rich in cellulose and lignin to the diet of pregnant sows significantly increased the pH value of feces, which might be related to its strong adsorption capability (Yang et al., 2010). Our study also found that feeding with 30 g d^−1^ and 60 g d^−1^ bamboo powder could significantly increase the pH value of the feces of perinatal sows. Since the feeding bamboo powder mainly included insoluble fiber, and it might absorb ammonia in the digestive tract, thus increasing the pH value of feces. However, another study found that the pH value in the fecal culture with a novel polysaccharide was decreased, and it might be related to modulating beneficial microbiota and the concentrations of fermentation products (Fu et al., 2018). Intestinal flora could secrete cellulolytic enzyme, which broke down glycosidic bonds and utilized carbohydrates, thus increasing the utilization rate of dietary fiber (Min et al., 2014). A study compared the application effects of lignocellulose, resistant starch, and konjac flour on sows in late pregnancy. The highest scores of defecation frequency and stool consistency were found in sows of the resistant starch group (Lu et al., 2022). The above results indicated that both fiber adsorption capacity and regulation of intestinal microbiota might affect fecal pH values. Fiber characteristics also affect fecal water content, it was found that adding either sugar beet pulp or wheat bran to the diet of sows could significantly improve the water content in feces, and beet pulp was better than bran in reducing inflammatory reactions and improving intestinal health (Shang et al., 2021a). Bamboo shoot dietary fiber could regulate the metabolism of the intestinal microbiota and the host, suggesting its use as a promising therapeutic strategy for ulcerative colitis (Wu et al., 2023). This study revealed that supplementary feeding of bamboo powder could significantly increase the water content of sow feces, and it was necessary to study its beneficial effects on intestinal health in the future. The above results indicated that supplementary feeding of bamboo powder for perinatal sows could improve blood lipid metabolism, antioxidation, and constipation, and 60 g d^−1^ was more effective than 30 g d^−1^.

### Effects of feeding bamboo powder on fecal microorganisms of sows

Microbial communities in animal intestines played an important role in physiological regulation, which might affect the intestinal structure and function through their metabolites (Clausen and Mortensen, 1995), and then influenced the nutrition, immunity, and physiological state of livestock and poultry (Ussar et al., 2016; Rubio, 2019). Dietary fiber increased the fecal microbiota richness (ACE) and diversity (Shannon and Simpson) in Meishan sows (Jiang et al., 2019). However, our study showed different results. Feeding sows with bamboo powder during the perinatal period reduced the fecal microbial diversity indexes (including Ace, Chao, and Sobs), which might be related to the fiber characteristics of the bamboo powder. The addition of different levels and types of dietary fiber were related to the gut microbiota abundance and diversity (Tian et al., 2020). Feeding bamboo powder was mainly made of insoluble fibers with a slow fermentation rate, which produced less short-chain fatty acids (SCFA) than that soluble fiber, thus reducing the richness of intestinal microflora (Jha and Berrocoso, 2015). Feeding bamboo powder reduced microbial diversity in sow feces, which might contribute to nutrient digestion and absorption. Feeding a high-fiber diet could improve the diversity and richness of microorganisms in the feces of finishing pigs, while it might also reduce the digestibility of amino acids and fats (Hu et al., 2021). Our study also found that supplementary feeding of bamboo powder in the perinatal period increased the fecal microbial diversity index of its suckling piglets. The results were similar to that of another study. Adding alfalfa to the diet of pregnant sows increased the diversity of intestinal flora of their piglets (Mu et al., 2017). The above results indicated that feeding bamboo powder in the perinatal period could regulate the microbial diversity in the feces of suckling piglets through the comprehensive regulation effects of sows, which might play beneficial roles for both sows and piglets.

A high-fiber diet could improve the intestinal balance and uniformity index of the intestinal flora of pregnant sows. Supplementing stevia residue in the basal diet of pregnant sows could improve the relative abundance of beneficial bacteria, reduce the relative abundance of harmful bacteria, and the feed cost could also be reduced (Yu et al., 2020). Adding 5% elephant grass to the late pregnancy diet might change the composition of intestinal microbiota of sows, especially increased the relative abundance of *Bacteroidetes* and *Actinobacteria* at phylum level and decreased the relative abundance of *Escherichia_Shigella* at genus level (Huang et al., 2021). Adding fibers to the diet of pregnant sows could improve the composition of gut microbiota, which might be the microbiological basis of its ability to improve the antioxidant and fecal water content. Our study revealed that the relative abundance of *Actinomyces* in sows was significantly reduced by supplementary feeding of bamboo powder in the perinatal period, which might be the basis of its influence on fecal microbiota of offspring piglets. It was showed that alterations in the composition of the gut microbiota by GCW (guar gum and pregelatinized waxy corn starch) treatment was associated with improved bile acid homeostasis and reproductive performance of sows (Wu et al., 2021). In another study, adding the mixture of pregelatinized waxy corn starch and guar gum (2%) in gestation diet increased the relative abundance of *Lactobacillus* in the feces of offspring piglets, which was positively related to the growth rate of piglets (Cheng et al., 2018). Feeding fiber for sows could significantly increase the microbiota diversity of colonic chyme of piglets, significantly elevate the abundance of *Acidobacteria* and *Bacteroidetes* at the phylum level, and the abundance of *Bradyrhizobium* and *Phyllobacterium* at the genus level (He et al., 2020). Therefore, it was necessary to study the effects of bamboo powder on regulating intestinal health and growth performance of offspring piglets from the perspective of maternal-offspring interactions.

Perinatal period of sows via from 7 to 10 days before delivery to 3–5 days after delivery. Although the perinatal period is short, it is very important for the productivity of sows. Most piglet deaths have happened during birth or within the 1st day of birth. After the farrowing, the breast development, fetal growth, farrowing process, and colostrum production of sows all have positive effects on the survival and growth of piglets. Nutrition in this perinatal period could greatly affect these physiological processes, and it is necessary to formulate the most effective feeding strategy. Feeding a high-fiber diet to perinatal sows from 7 days before delivery to 2 days after delivery could reduce *Clostridium perfringens* in feces, thus helping to improve the health of newborn piglets (Schwennen et al., 2022). Different fiber types have different effects on maternal-offspring interactions to regulate the microbiota of offspring piglets. In the group with a high ratio of insoluble fiber to soluble fiber, the fecal microbial diversity index of sows was lower, with higher abundance of *Streptococcus*, and the fecal microbial diversity index of offspring piglets was lower, with lower abundance of *Bifidobacterium* (Li et al., 2019). Compared with low fermentable fiber (lignocellulose), sows fed a diet rich in high fermentable fiber (sugar beet pulp) reduced the colonization of *Clostridium difficile* in piglets and the prevalence of *Clostridium difficile* in three-week-old piglets (Grześkowiak et al., 2022). This study revealed that the fecal microbial diversity of sows supplemented with feeding bamboo powder was reduced, while it was increased in offspring piglets. The relative abundance of *Actinobacteriota* in the feces of sows decreased significantly, while that of *Fusobacteriota* in the feces of piglets decreased. The relative abundance of *Tissierella* in the feces of sows decreased significantly, while that of *Fusobateriumv* in the feces of piglets decreased. This indicated that bamboo powder fiber might be different from soluble fiber in regulating the diversity and composition of microbiota *via* maternal-offspring interactions. For the diversity regulation, sows and piglets were not synchronized, but the trend was similar for decreasing the proportion of harmful bacteria, so it was necessary to further explore the relationship between the fiber characteristics of bamboo powder and the regulation of the gut microbiota and metabolism in sows and offspring piglets, and reveal why the application effects of bamboo powder differed with that of other fiber materials.

## Conclusion

Supplementary feeding of bamboo powder for sows in the perinatal period has no significant effect on the farrowing process. However, it could reduce the serum malondialdehyde content and increase the water content in the feces of sows. In addition, by feeding bamboo powder, fecal microbiotal diversity could be reduced in sows, while it was increased in suckling piglets, and the effect of supplementary feeding 60 g d^−1^ bamboo powder was more obvious. Feeding 60 g d^−1^ bamboo powder could reduce the abundance of *Fusobacterium* in the feces of offspring piglets, a pathogenic bacterium for suckling piglets. It plays an obvious role in improving oxidative damage and constipation of sows, and bacterial balance of offspring piglets. The obtained results indicate that feeding bamboo powder was expected to be a high-quality dietary fiber raw material for sows in the perinatal period.

## Data availability statement

The data presented in this study are deposited in the NCBI repository, accession number PRJNA953182.

## Ethics statement

The animal study was reviewed by Animal Care and Use Committee of Leshan Normal University (Certification No. 4151010649), China, and conducted in accordance with the approved protocol (No. LAC2022001). Written informed consent was obtained from the owners for the participation of their animals in this study.

## Author contributions

FD initiated the idea and finished the experiment design. FD, TL, XH, XS, and HL conducted the animal trial and laboratory index determination. TL and FD prepared the initial manuscript in English. YY, XN, and JZ made the final revision. All authors read and approved the final manuscript.

## Funding

This study was supported by the Key Program of Sichuan Science and Technology Plan (2021YFN0106) and Talent Start-up Project of Leshan Normal University (RC202004).

## Conflict of interest

XS and HL were employed by the company Beijing Vica Group Biotechnology Co., Ltd.

The remaining authors declare that the research was conducted in the absence of any commercial or financial relationships that could be construed as a potential conflict of interest.

## Publisher’s note

All claims expressed in this article are solely those of the authors and do not necessarily represent those of their affiliated organizations, or those of the publisher, the editors and the reviewers. Any product that may be evaluated in this article, or claim that may be made by its manufacturer, is not guaranteed or endorsed by the publisher.

## Footnotes

^1^ www.majorbio.com

^2^ https://github.com/OpenGene/fastp

^3^ http://www.cbcb.umd.edu/software/flash

^4^ http://drive5.com/uparse/

^5^ http://rdp.cme.msu.edu/

^6^ http://www.arb-silva.de

^7^ https://cloud.majorbio.com

^8^ http://www.mothur.org/wiki/Calculators

## References

[ref1] AOAC (2007). Official methods of analysis. 18th Edition, Association of Official Analytical chemists, Gaithersburg.

[ref2] Bakare A. G. Ndou S. P. Madzimure J. Chimonyo M. (2016). Nutritionally related blood metabolites and performance of finishing pigs fed on graded levels of dietary fibre. Trop. Anim. Health Prod. 48, 1065–1069. doi: 10.1007/s11250-016-1038-1, PMID: 26984596

[ref3] Basumatary A. Middha S. K. Usha T. Brahma B. K. Goyal A. K. J. R. I. P. B. (2015). Bamboo, as potential sources of food security, economic prosperity and ecological security in north-East India: an overview. Res. Plant Biol. 5, 17–23.

[ref4] Chen S. Zhou Y. Chen Y. Gu J. (2018). FASTP: an ultra-fast all-in-one FASTQ preprocessor. Bioinformatics 34, i884–i890. doi: 10.1093/bioinformatics/bty560, PMID: 30423086 PMC6129281

[ref5] Cheng C. Wei H. Xu C. Xie X. Jiang S. Peng J. (2018). Maternal soluble fiber diet during pregnancy changes the intestinal microbiota, improves growth performance, and reduces intestinal permeability in piglets. Appl. Environ. Microbiol. 84:e01047-18. doi: 10.1128/aem.01047-18, PMID: 29959248 PMC6102992

[ref6] Clausen M. R. Mortensen P. B. (1995). Kinetic studies on colonocyte metabolism of short chain fatty acids and glucose in ulcerative colitis. Gut 37, 684–689. doi: 10.1136/gut.37.5.684, PMID: 8549946 PMC1382875

[ref7] Dai F. Lin T. Cheng L. Wang J. Zuo J. Feng D. (2022). Effects of micronized bamboo powder on growth performance, serum biochemical indexes, cecal chyme microflora and metabolism of broilers aged 1-22 days. Trop. Anim. Health Prod. 54:166. doi: 10.1007/s11250-022-03172-0, PMID: 35437649 PMC9015971

[ref8] Dai F. Lin T. Su B. Yao H. Gu D. Yang Y. (2021). Effects of feeding bamboo powder on growth performance, serum biochemical indexes and fecal microorganism of weaned piglets. Chin. J. Anim. Nutr. 33, 6709–6720. doi: 10.3969/j.issn.1006-267×.2021.12.013

[ref9] Deng Y. F. Wang Y. J. Zou Y. Azarfar A. Wei X. L. Ji S. K.. (2017). Influence of dairy by-product waste milk on the microbiomes of different gastrointestinal tract components in pre-weaned dairy calves. Sci. Rep. 7:42689. doi: 10.1038/srep42689, PMID: 28281639 PMC5345013

[ref10] Edgar R. C. (2013). UPARSE: highly accurate OTU sequences from microbial amplicon reads. Nat. Methods 10, 996–998. doi: 10.1038/nmeth.2604, PMID: 23955772

[ref11] Felisberto M. H. F. Miyake P. S. E. Beraldo A. L. Clerici M. (2017). Young bamboo culm: potential food as source of fiber and starch. Food Res. Int. 101, 96–102. doi: 10.1016/j.foodres.2017.08.058, PMID: 28941702

[ref12] Feyera T. Højgaard C. K. Vinther J. Bruun T. S. Theil P. K. (2017). Dietary supplement rich in fiber fed to late gestating sows during transition reduces rate of stillborn piglets. J. Anim. Sci. 95, 5430–5438. doi: 10.2527/jas2017.2110, PMID: 29293780 PMC6292286

[ref13] Feyera T. Hu L. Eskildsen M. Bruun T. S. Theil P. K. (2021). Impact of four fiber-rich supplements on nutrient digestibility, colostrum production, and farrowing performance in sows. J. Anim. Sci. 99:skab247. doi: 10.1093/jas/skab247, PMID: 34420055 PMC8438544

[ref14] Fu X. Cao C. Ren B. Zhang B. Huang Q. Li C. (2018). Structural characterization and in vitro fermentation of a novel polysaccharide from *Sargassum thunbergii* and its impact on gut microbiota. Carbohydr. Polym. 183, 230–239. doi: 10.1016/j.carbpol.2017.12.048, PMID: 29352879

[ref15] Grześkowiak Ł. Saliu E. M. Martínez-Vallespín B. Wessels A. G. Männer K. Vahjen W.. (2022). Fiber composition in Sows' diets modifies *Clostridioides difficile* colonization in their offspring. Curr. Microbiol. 79:154. doi: 10.1007/s00284-022-02848-y, PMID: 35397071 PMC8994737

[ref16] He Y. Peng X. Liu Y. Wu Q. Zhou Q. Hu L.. (2020). Effects of maternal fiber intake on intestinal morphology, bacterial profile and proteome of newborns using pig as model. Nutrients 13:42. doi: 10.3390/nu13010042, PMID: 33375592 PMC7823571

[ref17] Hu P. Wang L. Hu Z. Jiang L. Hu H. Rao Z.. (2021). Effects of multi-bacteria solid-state fermented diets with different crude fiber levels on growth performance, nutrient digestibility, and microbial Flora of finishing pigs. Animals (Basel) 11:3079. doi: 10.3390/ani11113079, PMID: 34827811 PMC8614399

[ref18] Huang Y. Ji Y. Yu W. (2019). Development of bamboo scrimber: a literature review. J. Wood Sci. 65:25. doi: 10.1186/s10086-019-1806-4

[ref19] Huang P. F. Mou Q. Yang Y. Li J. M. Xu M. L. Huang J.. (2021). Effects of supplementing sow diets during late gestation with Pennisetum purpureum on antioxidant indices, immune parameters and faecal microbiota. Vet. Med. Sci. 7, 1347–1358. doi: 10.1002/vms3.450, PMID: 33620158 PMC8294372

[ref20] Jha R. Berrocoso J. D. (2015). Review: dietary fiber utilization and its effects on physiological functions and gut health of swine. Animal 9, 1441–1452. doi: 10.1017/s1751731115000919, PMID: 25997437 PMC4574174

[ref21] Jiang X. Lu N. Xue Y. Liu S. Lei H. Tu W.. (2019). Crude fiber modulates the fecal microbiome and steroid hormones in pregnant Meishan sows. Gen. Comp. Endocrinol. 277, 141–147. doi: 10.1016/j.ygcen.2019.04.006, PMID: 30951727

[ref22] Kohn R. A. Dinneen M. M. Russek-Cohen E. (2005). Using blood urea nitrogen to predict nitrogen excretion and efficiency of nitrogen utilization in cattle, sheep, goats, horses, pigs, and rats. J. Anim. Sci. 83, 879–889. doi: 10.2527/2005.834879x, PMID: 15753344

[ref23] Lanh D. T. K. Nam N. H. (2022). High stillbirth rate in a swine farm in Vietnam and associated risk factors. J. Adv. Vet. Anim. Res. 9, 13–18. doi: 10.5455/javar.2022.i564, PMID: 35445110 PMC8985879

[ref24] Li Y. Liu H. Zhang L. Yang Y. Lin Y. Zhuo Y.. (2019). Maternal dietary fiber composition during gestation induces changes in offspring Antioxidative capacity, inflammatory response, and gut microbiota in a sow model. Int. J. Mol. Sci. 21:31. doi: 10.3390/ijms21010031, PMID: 31861629 PMC6981455

[ref25] Li H. Ma L. Zhang L. Liu N. Li Z. Zhang F.. (2021). Dietary inulin regulated gut microbiota and improved neonatal health in a pregnant sow model. Front. Nutr. 8:716723. doi: 10.3389/fnut.2021.716723, PMID: 34434954 PMC8380823

[ref26] Li L. Pan M. Pan S. Li W. Zhong Y. Hu J.. (2020). Effects of insoluble and soluble fibers isolated from barley on blood glucose, serum lipids, liver function and caecal short-chain fatty acids in type 2 diabetic and normal rats. Food Chem. Toxicol. 135:110937. doi: 10.1016/j.fct.2019.110937, PMID: 31682932

[ref27] Li H. Song F. Duan L. R. Sheng J. J. Xie Y. H. Yang Q.. (2016). Paeonol and danshensu combination attenuates apoptosis in myocardial infarcted rats by inhibiting oxidative stress: roles of Nrf2/HO-1 and PI3K/Akt pathway. Sci. Rep. 6:23693. doi: 10.1038/srep23693, PMID: 27021411 PMC4810373

[ref28] Liu Y. Jiang P. Chen N. Jiang Y. Zhang R. Fang Z.. (2022). The improvement of parturition duration by high intake of dietary fibre in late gestation is associated with gut microbiota and metabolome in sows. Br. J. Nutr. 128, 2341–2352. doi: 10.1017/s0007114522000502, PMID: 35152932

[ref29] Liu C. Zhao D. Ma W. Guo Y. Wang A. Wang Q.. (2016). Denitrifying sulfide removal process on high-salinity wastewaters in the presence of *Halomonas* sp. Appl. Microbiol. Biotechnol. 100, 1421–1426. doi: 10.1007/s00253-015-7039-6, PMID: 26454867

[ref30] Lu D. Pi Y. Ye H. Wu Y. Bai Y. Lian S.. (2022). Consumption of dietary fiber with different physicochemical properties during late pregnancy alters the gut microbiota and relieves constipation in sow model. Nutrients 14(12):2511. doi: 10.3390/nu14122511, PMID: 35745241 PMC9229973

[ref31] Magoč T. Salzberg S. L. (2011). FLASH: fast length adjustment of short reads to improve genome assemblies. Bioinformatics 27, 2957–2963. doi: 10.1093/bioinformatics/btr507, PMID: 21903629 PMC3198573

[ref32] Min F. F. Hu J. L. Nie S. P. Xie J. H. Xie M. Y. (2014). In vitro fermentation of the polysaccharides from Cyclocarya paliurus leaves by human fecal inoculums. Carbohydr. Polym. 112, 563–568. doi: 10.1016/j.carbpol.2014.06.027, PMID: 25129782

[ref33] Mu C. Zhang L. He X. Smidt H. Zhu W. (2017). Dietary fibres modulate the composition and activity of butyrate-producing bacteria in the large intestine of suckling piglets. Antonie Van Leeuwenhoek 110, 687–696. doi: 10.1007/s10482-017-0836-4, PMID: 28161736

[ref34] Muller T. L. Pluske J. R. Plush K. J. D'Souza D. N. Miller D. W. van Barneveld R. J. (2022). Serum creatinine is a poor marker of a predicted change in muscle mass in lactating sows. J. Anim. Physiol. Anim. Nutr. (Berl.) 106, 1009–1016. doi: 10.1111/jpn.13637, PMID: 34528730

[ref35] Ndou S. P. Kiarie E. Thandapilly S. J. Walsh M. C. Ames N. Nyachoti C. M. (2017). Flaxseed meal and oat hulls supplementation modulates growth performance, blood lipids, intestinal fermentation, bile acids, and neutral sterols in growing pigs fed corn-soybean meal-based diets. J. Anim. Sci. 95, 3068–3078. doi: 10.2527/jas.2016.1328, PMID: 28727078

[ref36] Oh S. Hosseindoust A. Ha S. Moturi J. Mun J. Tajudeen H.. (2022). Metabolic responses of dietary fiber during heat stress: effects on reproductive performance and stress level of gestating sows. Meta 12:280. doi: 10.3390/metabo12040280, PMID: 35448467 PMC9028640

[ref37] Papatsiros V. G. Katsarou M. S. Drakoulis N. Maragkakis G. Tzika E. Maes D.. (2021). Effects of dietary fibre on metabolism and performance in sows. Pol. J. Vet. Sci. 24, 271–279. doi: 10.24425/pjvs.2021.137662, PMID: 34250787

[ref38] Pi Y. Hu J. Bai Y. Wang Z. Wu Y. Ye H.. (2021). Effects of dietary fibers with different physicochemical properties on fermentation kinetics and microbial composition by fecal inoculum from lactating sows in vitro. J. Sci. Food Agric. 101, 907–917. doi: 10.1002/jsfa.10698, PMID: 32737882

[ref39] Qi M. Nelson K. E. Daugherty S. C. Nelson W. C. Hance I. R. Morrison M.. (2005). Novel molecular features of the fibrolytic intestinal bacterium *Fibrobacter intestinalis* not shared with *Fibrobacter succinogenes* as determined by suppressive subtractive hybridization. J. Bacteriol. 187, 3739–3751. doi: 10.1128/jb.187.11.3739-3751.2005, PMID: 15901698 PMC1112041

[ref40] Ren W. Yin Y. Liu G. Yu X. Li Y. Yang G.. (2012). Effect of dietary arginine supplementation on reproductive performance of mice with porcine circovirus type 2 infection. Amino Acids 42, 2089–2094. doi: 10.1007/s00726-011-0942-y, PMID: 21617969 PMC3351591

[ref41] Rubio L. A. (2019). Possibilities of early life programming in broiler chickens via intestinal microbiota modulation. Poult. Sci. 98, 695–706. doi: 10.3382/ps/pey416, PMID: 30247675

[ref42] Schwennen C. Reckels B. Klingenberg M. El-Wahab A. A. Keller B. Visscher C. (2022). Influence of feeding compound feed rich in fibre during parturition and lactation on health and performance of sows. Animals (Basel) 12(4), 497. doi: 10.3390/ani12040497, PMID: 35203205 PMC8868540

[ref43] Shang Q. Liu S. Liu H. Mahfuz S. Piao X. (2021a). Impact of sugar beet pulp and wheat bran on serum biochemical profile, inflammatory responses and gut microbiota in sows during late gestation and lactation. J. Anim. Sci. Biotechnol. 12:54. doi: 10.1186/s40104-021-00573-3, PMID: 33879267 PMC8059298

[ref44] Shang Q. Liu S. Liu H. Mahfuz S. Piao X. (2021b). Maternal supplementation with a combination of wheat bran and sugar beet pulp during late gestation and lactation improves growth and intestinal functions in piglets. Food Funct. 12, 7329–7342. doi: 10.1039/d1fo00014d, PMID: 34179915

[ref45] Stackebrandt E. Goebel B. M. (1994). Taxonomic note: a place for DNA-DNA Reassociation and 16S rRNA sequence analysis in the present species definition in bacteriology. Int. J. Syst. Evol. Micr. 44, 846–849. doi: 10.1099/00207713-44-4-846

[ref46] Tabeling R. Schwier S. Kamphues J. (2003). Effects of different feeding and housing conditions on dry matter content and consistency of faeces in sows. J. Anim. Physiol. Anim. Nutr. (Berl.) 87, 116–121. doi: 10.1046/j.1439-0396.2003.00423.x, PMID: 14511136

[ref47] Theil P. K. Farmer C. Feyera T. (2022). Review: physiology and nutrition of late gestating and transition sows. J. Anim. Sci. 100:skac176. doi: 10.1093/jas/skac176, PMID: 35708593 PMC9202569

[ref48] Tian M. Chen J. Liu J. Chen F. Guan W. Zhang S. (2020). Dietary fiber and microbiota interaction regulates sow metabolism and reproductive performance. Anim Nutr. 6, 397–403. doi: 10.1016/j.aninu.2020.10.001, PMID: 33364455 PMC7750804

[ref49] Ussar S. Fujisaka S. Kahn C. R. (2016). Interactions between host genetics and gut microbiome in diabetes and metabolic syndrome. Mol Metab 5, 795–803. doi: 10.1016/j.molmet.2016.07.004, PMID: 27617202 PMC5004229

[ref50] Van Soest P. J. Robertson J. B. Lewis B. A. (1991). Methods for dietary fiber, neutral detergent fiber, and nonstarch polysaccharides in relation to animal nutrition. J. Dairy Sci. 74, 3583–3597. doi: 10.3168/jds.S0022-0302(91)78551-2, PMID: 1660498

[ref51] Wang Q. Garrity G. M. Tiedje J. M. Cole J. R. (2007). Naive Bayesian classifier for rapid assignment of rRNA sequences into the new bacterial taxonomy. Appl. Environ. Microbiol. 73, 5261–5267. doi: 10.1128/aem.00062-07, PMID: 17586664 PMC1950982

[ref52] Wang Y. Nan X. Zhao Y. Jiang L. Wang H. Zhang F.. (2021). Dietary supplementation of inulin ameliorates subclinical mastitis via regulation of rumen microbial community and metabolites in dairy cows. Microbiol. Spectr. 9:e0010521. doi: 10.1128/Spectrum.00105-21, PMID: 34494854 PMC8557905

[ref53] Weng R. C. (2020). Dietary supplementation with different types of fiber in gestation and lactation: effects on sow serum biochemical values and performance. Asian-Australas J. Anim. Sci. 33, 1323–1331. doi: 10.5713/ajas.19.0545, PMID: 32054223 PMC7322642

[ref54] Wu W. Hu J. Gao H. Chen H. Fang X. Mu H.. (2020). The potential cholesterol-lowering and prebiotic effects of bamboo shoot dietary fibers and their structural characteristics. Food Chem. 332:127372. doi: 10.1016/j.foodchem.2020.127372, PMID: 32615381

[ref55] Wu W. Li Q. Chen H. Fang X. Niu B. Liu R.. (2023). In vitro fermentation characteristics of the dietary fiber in bamboo (*Phyllostachys edulis*) shoots and its regulatory effects on the intestinal microbiota and metabolites. Food Chem. 404:134707. doi: 10.1016/j.foodchem.2022.134707, PMID: 36327509

[ref56] Wu X. Yin S. Cheng C. Xu C. Peng J. (2021). Inclusion of soluble fiber during gestation regulates gut microbiota, improves bile acid homeostasis, and enhances the reproductive performance of sows. Front. Vet. Sci. 8:756910. doi: 10.3389/fvets.2021.756910, PMID: 34869730 PMC8635514

[ref57] Xie L. M. Ge Y. Y. Huang X. Zhang Y. Q. Li J. X. (2015). Effects of fermentable dietary fiber supplementation on oxidative and inflammatory status in hemodialysis patients. Int. J. Clin. Exp. Med. 8, 1363–1369. PMID: 25785138 PMC4358593

[ref58] Yang Y. Ge D. Wang C. (2010). Effects of dietary fiber levels on fecal indices and serum hormones and other biochemical indices of gestating sows. Chin. J. Anim. Nutr. 22, 1529–1535. doi: 10.3969/j.issn.1006-267x.2010.06.010

[ref59] Yu M. Gao T. Liu Z. Diao X. (2020). Effects of dietary supplementation with high fiber (stevia residue) on the fecal Flora of pregnant sows. Animals (Basel) 10(12), 2247. doi: 10.3390/ani10122247, PMID: 33266059 PMC7761306

